# Muscle assessment using multi‐frequency bioimpedance in a healthy Danish population aged 20–69 years: a powerful non‐invasive tool in sports and in the clinic

**DOI:** 10.14814/phy2.14109

**Published:** 2019-06-13

**Authors:** Else Marie Bartels, Eva Littrup Andersen, Jack Kvistgaard Olsen, Lars Erik Kristensen, Henning Bliddal, Bente Danneskiold‐Samsøe, Adrian Paul Harrison

**Affiliations:** ^1^ The Parker Institute Copenhagen University Hospital Bispebjerg & Frederiksberg Frederiksberg Denmark; ^2^ Department of Neurology Copenhagen University Hospital Bispebjerg & Frederiksberg Copenhagen Denmark; ^3^ Copenhagen Center for Translational Research Copenhagen University Hospital, Bispebjerg & Frederiksberg Copenhagen Denmark; ^4^ Department of Clinical Medicine Faculty of Health & Medical Sciences Copenhagen University Copenhagen Denmark; ^5^ Pathobiological Sciences (Physiology) Faculty of Health & Medical Sciences Copenhagen University Frederiksberg Denmark

**Keywords:** Health, impedance, multi‐frequency bioimpedance, muscle mass, skeletal muscle

## Abstract

The condition of active muscles determines an individual′s ability to carry out daily activities and has implications for an athlete′s performance. Multi‐frequency bioimpedance (mfBIA) is a non‐invasive, well‐known, validated, and much used method to assess muscle condition. However, it is rarely used to its full potential. Our aim was to apply mfBIA fully in the assessment of an adult healthy population, to compare muscle condition in different functional rested muscle groups, with age, and between men and women, and establish a control data set. Fifty healthy subjects (25 men/25 women) aged 20–69 years, participated. mfBIA measurements at a frequency range of 4–1000 kHz were taken from muscles of the lower and the upper extremities, the upper back, and the hand. Data were analyzed using ImpediMed software, giving Impedance, Resistance, Reactance, Phase Angle, Center Frequency, external and internal Resistance, and Membrane Capacitance. Differences between means were tested for statistical significance. A *P* value >0.05 was considered nonsignificant. While no difference in the mfBIA parameters was seen with age, a highly significant gender difference was seen. At rest, women′s muscles cf men's showed a significantly higher center frequency and intra‐ and extra‐cellular resistance, while the membrane capacitance was lower. A set of values for mfBIA parameters for healthy adult individuals are given for some of the main muscles which are frequently part of muscle assessment. The documented gender difference in muscle condition at rest has important implications in work situations, during physical rehabilitation and when training for competitive sports.

## Introduction

The condition of involved muscles when wishing to carry out a task, such as lifting a weight or simply walking, is important to a subject's stamina and ability to carry out daily activities, as well as for an athlete's performance success.

An individual's muscle mass and composition are decided by genetic base, age and environment (van Praagh and Doré 2002; Kasper 2011; Costa et al. 2012; Landi et al. 2012, 2015; Garatachea and Lucia 2013; Giordani and Puri 2013), presence of musculoskeletal diseases (Felin et al. 2007; Henriksen et al. 2009; Winters and Rudolph 2014; Colombo et al. 2015; Davis et al. 2015; Hall et al. 2015; Jones and Wortmann 2015), as well as by training/fitness level (Fiatarone et al. 1990; Bousquet‐Santos et al. 2006; Arnold and Bautmans 2014; Chung et al. 2015). Prior to carrying out a muscle task, it is therefore useful to know the condition of the muscle in question. In many movements like walking, involving both anatomical halves (left‐side vs. right‐side) of the body, it is well known that specific myofascial kinetic lines are activated (Stecco et al. 2013). It is also important to know the balance between the left‐side and the right‐side muscles to assure not only a stabilizing role of these muscles, but also a balanced power stroke. In other movements like bending of the elbow, the condition of the agonist and antagonist muscles likewise influences movement. For the purpose of assessing muscle condition prior to movement, multi‐frequency bioimpedance (mfBIA) (de Luca 1997; Hermens et al. 1999) which is a well‐known, validated, and much used method in both sports clubs and in the clinic, may be used (Gerdle et al. 1997; Bernardi et al. 1999; Karlsson and Gerdle 2001; Bajaj et al. 2002; Larsson et al. 2006; Moon et al. 2008; Harrison et al. 2012, 2013; Lukaski 2013). mfBIA has been shown to characterize the relative changes in hydration as well as monitor changes in cellular function in a noninvasive way (Nescolarde et al. 2013a,2013b). The principle values obtained are Impedance (Z), Resistance (R), Reactance (Xc), Phase Angle (PA), Center Frequency (fc), external (Re) and internal Resistance (Ri), and Membrane Capacitance (Mc). R, is the ohmic resistance and Z the impedance of a measured tissue, which in uniform muscle are very comparable values (Z slightly higher than R) (Ivorra 2003; Van der Aa Kuhle et al. 2006). Xc, measures the delay in the current passing through the cell membrane and tissue interfaces (Ivorra 2003). Fc provides a measure for how dense a muscle is at rest (Elbrønd et al. 2015). Mc gives an indication of transport activity over the muscle cell membrane, thereby telling about metabolic activity in the cell, but it is also very much related to the thickness of the cell membrane (Ivorra 2003; Dodde et al. 2012; Tan et al. 2012). Ri, has been directly related to VOmax, the maximal aerobic capacity, and thereby oxygen consumption of the muscle cells (Stahn et al. 2006, 2008). Mc and Ri together will therefore give an indication of metabolic activity without any invasive and tedious measurements. Re and R provide an insight into the local hydration of a muscle, high values representing a relatively hypotonic state around the muscle cells (Ivorra 2003), while low values are associated with injury and swollen tissue (Nescolarde et al. 2013b, 2015). Phase angle has been used as a means of assessing changes in individuals with different body mass, representing a ratio of R and Xc (Ivorra 2003; Bartels et al. 2015).

The overall aim of this study was to apply mfBIA fully in the assessment of an adult healthy population, to compare muscle condition in different functional rested muscle groups, with age, and between men and women, thereby establishing a control data set. The hypotheses tested were therefore; (1) age affects muscle fiber condition; and (2) there is a gender difference in the physiological condition of resting muscle.

## Materials and Methods

### Ethics approval

The methods applied were non‐invasive apart from a standard blood test. The study followed the guidelines set by the Helsinki Declaration 2013 (http://www.wma.net/en/30publications/10policies/b3/), and the subjects gave their informed written consent prior to participating in this study. The data set comprises part of a much larger study on physical performance in healthy adults. The study was approved by the Capital Region of Denmark's Ethics Committee (H‐15017787) and was registered with the Danish Data Protection Agency.

### Subjects

10 healthy subjects, 5 men and 5 women, in each age decade from 20 to 69 years of age, in total 50 subjects, participated in this study. Recruitment happened via advertising and word of mouth. Measurements took place at the Parker Institute, Copenhagen University Hospital, Bispebjerg and Frederiksberg, Denmark. Inclusion criteria for this study were, (1) age between 20 and 69 years; (2) 18.5 < Body Mass Index (BMI) < 30; (3) healthy according to assessment by a series of blood test and a physical examination by a physician (see Table 1) prior to measurements (analyses carried out at Bispebjerg & Frederiksberg Hospitals, Clinical Chemistry Dept, as routine tests); (4) no chronic or present illness; (5) no intake of medicine except birth control pills; and (5) pain reported in the normal range and pattern for healthy subjects through answers to the PainDETECT Questionnaire (PD‐Q) ©2005Pfizer Pharma GmbH (Freynhagen et al. 2006) prior to participation.

**Table 1:** A list of the blood tests taken for the assessment of the participants as being healthy individuals

1	Glycated Hamoglobin‐c (HbA1c)
2	Alanine‐transaminase
3	Alkaline Phosphatase
4	C‐Reactive Protein (CRP)
5	Erythrocytes
6	Erythrocyte volume fraction (EVOL)
7	Erythrocyte volume Mean (MCV)
8	Hamoglobin (Hb)
9	Mean corpuscular hamoglobin concentration (MCHC)
10	Potassium
11	Creatinine
12	Leucocytes
13	Leucocyte type and group (DIFFMAS)
14	Sodium
15	Reticulocyte group
16	Thrombocytes

Subjects were excluded if they were outside the defined age range or could not understand instructions in the Danish language, or indeed did not comply with the inclusion criteria.

### Measurements

mfBIA measurements were taken from the lower extremities; *m. Gastrocnemius, m. Rectus femoris, m. Tibialis anterior* and *m*. *Vastus lateralis,* as well as from the upper extremities; *m. Biceps and m. Triceps,* and from the upper back; *m. Trapezius*. In addition, *m. Abductor pollicis brevis* of the hand was also measured.

The mfBIA measurements were undertaken using an ImpediMed Inc tetra‐polar bioimpedance spectroscopy unit (Impedimed, Pinkenba, Qld, Australia) with matching ImpediMed electrodes according to the firm's recommendations. These measurements were made at rest, that is the subject arrived without having carried out strenuous physical activity for 24 h prior to measurement.

mfBIA measurements were made in a standing position, ensuring that the subject was kept free of all metal and human contact. In each case, the four electrodes were placed onto the identified muscle sites, two at the insertion and two at the origin, as documented as being optimal (Nescolarde et al. 2013a,2013b). For all electrode placements, the outer two electrodes provided the electrical field (Alternating Current), and the inner pair was used as sensing electrodes (Voltage). Measurements were carried out over a frequency range of 4–1000 kHz, applying 256 different frequencies which were plotted in real‐time as a Cole‐Cole plot (Ivorra 2003; Grimnes and Martinsen 2008). These values, as well as the Cole‐Cole plot, could be seen for each subject as they were made, ensuring accurate data collection.

The overall sequence of testing involved (1) answering the PainDetect Questionnaire (Freynhagen et al. 2006) and a general health questionnaire concerning medicine and chronic disease; (2) a blood test, as well as weight, height and handedness measurement; (3) acceptance as being healthy by a clinician; and (4) mfBIA measurements at rest.

### Data handling

The bioimpedance data were analyzed using the ImpediMed Inc software (Impedimed, Pinkenba, Qld, Australia). Initially, the Cole‐Cole plot was analyzed to assess its normal distribution, and subsequently both the R and Xc plots were examined to ensure that a correct recording had been obtained. Following this, the Center Frequency (fc) and the Extracellular Resistance (Re) were determined from the Cole‐Cole plot, and intracellular Ri was calculated from the formula: Ri = (Re × R∞/Re‐R∞). Membrane Capacitance (M_c_) was also calculated from the formula: fc = 1/(2*π* × Mc × (Re + Ri). A detailed analysis was finally performed at 50 kHz with measurement of Resistance (R) and Reactance (Xc), and a calculation of the Impedance (Z), where Z = Square Root (R^2^ + Xc^2^). Finally, in accordance with other studies (Bartels et al. 2015; Harrison et al. 2015), and so as to be able to compare between individuals of different body mass, the Phase Angle (PA) was calculated: PA = arctan (Xc/R) with units in degrees. The mfBIA parameters were interpreted in terms of muscle mass (Z, R), energy storage capacity/fiber size (Xc), hydration status (R, Re), tissue density/resting tension (fc), membrane activity/integrity (Mc) and metabolic activity (Ri) (Bartels et al. 2015; Harrison et al. 2015).

### Statistical analysis

Changes in mfBIA parameters with age were tested by comparing the two youngest groups (years 20 + 30) pooled with the two oldest groups (years 50 + 60) pooled for men and women, respectively. This was done to get enough power, since we were limited to 50 participants in this study. Data were initially tested for a normal distribution and equal variance. Subsequently data were analyzed using a two‐tailed unpaired *t*‐test (Armitage et al. 2001) when normally distributed. Where normal distribution was not found, a Mann–Whitney test (Mann and Whitney 1947) was applied.

Gender differences of the mfBIA parameters were tested using the Mann–Whitney test (Mann and Whitney 1947) since a few of the data sets were not found to be normally distributed. Where percentage change is shown, the mean value for the men was taken as being the baseline.

The statistical software used was GraphPad InStat 3 for Mac (Version 3.0b, 2003; Graph‐ Pad Inc., La Jolla, CA). A *P* value >0.05 was considered nonsignificant. Values are presented as mean ± the standard deviation of the mean.

## Results

### Population

An overview of the 50 healthy subjects can be seen in Table 2, where their age, height, weight and body mass index (BMI) are detailed. All the blood tests showed values in the normal range, as did the pain detect questionnaire.

**Table 2:** A description of the healthy population participating in this study. All values are given as mean ± standard deviation (SD). Weight is given in kilograms (kg) and height in meters (m)

Age group	Men				Women			
	Age (years)	Weight (kg)	Height (m)	BMI	Age (years)	Weight (kg)	Height (m)	BMI
20–29 years	28 ± 1	90 ± 1	1.86 ± 0.07	26.0 ± 1.7	25 ± 4	64 ± 1	1.71 ± 0.06	21.8 ± 1.1
30–39 years	35 ± 3	90 ± 11	1.85 ± 0.04	26.0 ± 2.8	35 ± 5	63 ± 6	1.69 ± 0.07	22.0 ± 4.3
40–49 years	44 ± 5	90 ± 8	1.83 ± 0.01	26.8 ± 2.2	44 ± 3	62 ± 1	1.65 ± 0.06	22.7 ± 2.0
50–59 years	53 ± 3	83 ± 19	1.81 ± 0.10	25.5 ± 3.2	54 ± 2	67 ± 11	1.69 ± 0.04	23.4 ± 4.1
60–69 years	65 ± 3	72 ± 8	1.76 ± 0.03	23.3 ± 2.7	62 ± 2	69 ± 18	1.65 ± 0.03	25.4 ± 5.4

### mfBIA measurements

The parameters measured in this study were: Impedance (Z), Resistance (R), Center Frequency (fc), Phase Angle (PA), Intracellular Resistance (Ri), Membrane Capacitance (Mc), Extracellular Resistance (Re), and Reactance (Xc).

### The effect of age on mfBIA parameters

Due to the limited number of participants, the effect of age was tested by comparing the pooled data from the two youngest groups against the pooled data from the two oldest groups. For the gastrocnemius muscle there were measurements for both left and right legs. The data therefore represent 20 recordings in each group.

Table 3 shows the effect of age on the mfBIA parameters for all eight of the measured muscles. No significant effect of age was seen for any of the mfBIA parameters in any of the muscles, despite their diverse functions. This allowed us to pool data from all women and compare them with data from all men when looking at gender differences.

**Table 3:** mfBIA parameters analyzed for significance with regards to gender (men vs. women) and with regard to age (20+30 years vs. 50+60 years)

	Z	R	fc	PA	Ri	Re	Xc	Mc
Arm								
*m. Biceps*								
Women versus men	↑54% *P* < 0.001	↑56% *P* < 0.001	↑37% *P* < 0.001	↓44% *P* < 0.001	↑164% *P* < 0.001	↑36% *P* < 0.001	↓13% *P* < 0.05	↓63% *P* < 0.001
20 + 30 versus 50 + 60	NS	NS	NS	NS	NS	NS	NS	NS
* m*. *Triceps*								
Women versus men	↑50% *P* < 0.001	↑51% *P* < 0.001	NS	↓46% *P* < 0.001	↑121% *P* < 0.001	↑48% *P* < 0.01	↓18% *P* < 0.05	↓27% NS
20 + 30 versus 50 + 60	NS	NS	NS	NS	NS	NS	NS	NS
Hand								
*m. Abductor pollicis*								
Women versus men	NS	NS	↑30% *P* < 0.001	↓17% *P* < 0.001	NS	NS	NS	↓35% *P* < 0.001
20 + 30 versus 50 + 60	NS	NS	NS	NS	NS	NS	NS	NS
Shoulder								
*m. Trapezius*								
Women versus men	↑15% *P* < 0.01	↑16% *P* < 0.001	↑40% *P* < 0.001	↓16% *P* < 0.05	↑33% *P* < 0.05	↑8% *P* < 0.05	NS	↓27% *P* < 0.001
20 + 30 versus 50 + 60	NS	NS	NS	NS	NS	NS	NS	NS
Leg								
*m*. *Rectus femoris*								
Women versus Men	↑69% *P* < 0.001	↑71% *P* < 0.001	↑62% *P* < 0.001	↓52% *P* < 0.001	↑190% *P* < 0.001	↑50% *P* < 0.001	↓14% *P* < 0.01	↓45% *P* < 0.05
20 + 30 versus 50 + 60	NS	NS	NS	NS	NS	NS	NS	NS
*m*. *Vastus lateralis*								
Women versus men	↑80% *P* < 0.001	↑85% *P* < 0.001	↑17% *P* < 0.01	↓63% *P* < 0.001	↑311% *P* < 0.001	↑48% *P* < 0.001	↓32% *P* < 0.001	↓69% *P* < 0.001
20 + 30 versus 50 + 60	NS	NS	NS	NS	NS	NS	NS	NS
*m*. *Gastrocnemius*								
Women versus men	↑49% *P* < 0.001	↑52% *P* < 0.001	↑26% *P* < 0.01	↓47% *P* < 0.001	↑197% *P* < 0.001	↑36% *P* < 0.001	↓22% *P* < 0.001	↓46% *P* < 0.01
20 + 30 versus 50 + 60	NS	NS	NS	NS	NS	NS	NS	NS
*m*. *Tibialis anterior*								
Women versus men	↑53% *P* < 0.001	↑57% *P* < 0.001	↑23% *P* < 0.01	↓43% *P* < 0.001	↑204% *P* < 0.001	↑36% *P* < 0.001	↓16% *P* < 0.01	↓46% *P* < 0.001
20 + 30 versus 50 + 60	NS	NS	NS	NS	NS	NS	NS	NS

### The effect of gender on mfBIA parameters

It was found that for all the muscles included in this study there were highly significant changes in the mfBIA parameters with regards to gender (Table 3).

The center frequency (fc), extracellular resistance (Re), intracellular resistance (Ri), resistance (R) and impedance (Z) were found on average to be significantly 33%, 37%, 174%, 55%, and 52% higher in women than in men, respectively. However, the reactance (Xc), membrane capacitance (Mc) and phase angle (PA) were found to be on average 19%, 44% and 41% lower in women than in men, respectively.

### Reference data for healthy adult subjects for the mfBIA parameters

For reference purposes, the mfBIA values for each muscle and for both men and women are listed below as mean ± SD where *n* = 25 for each value. Z, R, Xc, Ri and Re are given in Ω; fc is given in kHz; PA is given in (°); Mc is given in pF.

### Arm muscles

#### *m. Biceps*

The mfBIA values for men were as follows; Z 86.4 ± 19.2; R 84.2 ± 20.2; fc 42.3 ± 6.4; PA 13.1 ± 5.1; Ri 140.8 ± 84.6; Re 114.0 ± 19.2; Xc 18.1 ± 3.7; Mc 17.5 ± 7.6. The mfBIA values for women were as follows; Z 133.0 ± 27.6; R 131.9 ± 28.1; fc 58.1 ± 8.0; PA 7.3 ± 3.1; Ri 372.4 ± 211.7; Re 154.9 ± 26.9; Xc 15.7 ± 4.,3; Mc 6.4 ± 3.0.

#### *m. Triceps*

The mfBIA values for men were as follows; Z 102.8 ± 19.4; R 101.4 ± 20.1; fc 30.9 ± 6.3; PA 9.1 ± 3.9; Ri 258.9 ± 123.0; Re 131.8 ± 18.5; Xc 15.2 ± 4.1; Mc 15.5 ± 6.6. The mfBIA values for women were as follows; Z 154.1 ± 31.2; R 153.5 ± 31.4; fc 63.3 ± 95.4; PA 4.8 ± 2.1; Ri 573.4 ± 288.4; Re 195.9 ± 116.0; Xc 12.5 ± 4.1; Mc 11.3 ± 28.6.

### Hand muscle

#### *m. Abductor pollicis brevis*

The mfBIA values for men were as follows; Z 42.8 ± 6.4; R 42.2 ± 6.2; fc 73.5 ± 14.1; PA 8.8 ± 1.9; Ri 78.6 ± 20.7; Re 50.6 ± 9.1; Xc 6.6 ± 2.0; Mc 17.8 ± 4.0. The mfBIA values for women were as follows; Z 52.2 ± 25.8; R 51.7 ± 25.8; fc 95.9 ± 19.8; PA 7.3 ± 1.9; Ri 133.9 ± 214.7; Re 59.2 ± 27.1; Xc 6.3 ± 1.9; Mc 11.6 ± 3.7.

### Shoulder muscle

#### *m*. *Trapezius*

The mfBIA values for men were as follows; Z 71.7 ± 15.4; R 70.4 ± 15.9; fc 45.1 ± 7.2; PA 10.6 ± 4.6; Ri 144.5 ± 90.9; Re 90.8 ± 15.1; Xc 12.3 ± 3.6; Mc 18.2 ± 8.5. The mfBIA values for women were as follows; Z 82.7 ± 17.4; R 81.7 ± 17.8; fc 63.2 ± 18.9; PA 8.9 ± 3.7; Ri 193.0 ± 148.0; Re 98.5 ± 17.7; Xc 12.0 ± 3.7; Mc 13.3 ± 12.1.

### Leg muscles

#### *m. Rectus femoris*

The mfBIA values for men were as follows; Z 83.1 ± 16.1; R 81.7 ± 16.6; fc 33.2 ± 4.2; PA 10.5 ± 3.5; Ri 173.9 ± 73.5; Re 108.5 ± 17.0; Xc 14.4 ± 2.5; Mc 18.8 ± 6.0. The mfBIA values for women were as follows; Z 140.9 ± 19.2; R 140.4 ± 19.1; fc 53.7 ± 69.3; PA 5.0 ± 1.2; Ri 505.1 ± 164.2; Re 163.3 ± 23.9; Xc 12.3 ± 3.4; Mc 10.2 ± 19.7.

#### *m. Vastus lateralis*

The mfBIA values for men were as follows; Z 68.9 ± 15.9; R 66.6 ± 16.4; fc 31.8 ± 4.2; PA 15.1 ± 4.4; Ri 106.1 ± 65.7; Re 98.2 ± 18.3; Xc 17.1 ± 3.4; Mc 27.4 ± 7.8. The mfBIA values for women were as follows; Z 124.0 ± 23.0; R 123.4 ± 23.0; fc 37.3 ± 8.4; PA 5.5 ± 2.0; Ri 436.7 ± 155.6; Re 146.0 ± 27.2; Xc 11.6 ± 3.9; Mc 8.4 ± 3.6.

#### *m. Gastrocnemius*

The mfBIA values for men were as follows; Z 68.2 ± 13.4; R 66.6 ± 13.3; fc 52.5 ± 7.9; PA 12.2 ± 2.8; Ri 100.3 ± 38.1; Re 85.6 ± 16.9; Xc 14.2 ± 3.8; Mc 17.7 ± 4.8. The mfBIA values for women were as follows; Z 102.1 ± 20.6; R 101.5 ± 20.6; fc 66.5 ± 20.3; PA 6.4 ± 1.7; Ri 298.5 ± 116.7; Re 116.6 ± 22.4; Xc 11.1 ± 2.8; Mc 9.5 ± 13.9.

#### *m. Tibialis anterior*

The mfBIA values for men were as follows; Z 59.3 ± 8.0; R 57.5 ± 7.8; fc 48.4 ± 5.2; PA 14.0 ± 1.8; Ri 72.8 ± 18.4; Re 78.6 ± 10.6; Xc 14.3 ± 2.4; Mc 22.6 ± 4.5. The mfBIA values for women were as follows; Z 91.1 ± 20.5; R 90.2 ± 20.6; fc 59.7 ± 16.3; PA 7.9 ± 2.5; Ri 221.7 ± 105.7; Re 107.4 ± 22.3; Xc 12.1 ± 2.8; Mc 12.2 ± 12.8.

## Discussion

To the best of our knowledge this is the first study of its kind to assess muscle condition with mfBIA of a number of diverse skeletal muscles in healthy subjects of both genders, spanning an age range of 20–69 years, the normal span of most individuals’ working life.

### Effect of age on mfBIA parameters and the condition of the muscles

With age, our hypothesis was that muscle condition changed with increasing years. Contrary to this, in both men and women, age did not show an effect on mfBIA values for muscles in this healthy population aged over the range 20–69 years. Looking at the parameters describing a healthy structure of a muscle, Z and R, these indicate that although whole muscle atrophy may occur with increasing age, and it is known that muscle strength (Danneskiold‐Samsoe et al. 2009) decreases with age, the muscle fibers comprising the individual muscles are in themselves structurally healthy. This is further supported by the fact that both the Xc and the PA values describing fiber size and/or cellular energy storage (Tonkovic et al. 2000; Lukaski 2013; Nescolarde et al. 2013a), thereby expressing general cellular health, remain unchanged. There were no signs of differences in resting tension (fc) with age (Elbrønd et al. 2015), nor any difference in membrane capacitance (Mc), or in the intracellular resistance (Ri), which has been shown to be associated with cellular oxygen use (metabolism) (Stahn et al. 2006, 2008).

### Effect of gender on mfBIA parameters and the condition of the muscles

We hypothesized that there would be a gender difference in the physiological condition of resting muscle. Our findings did indeed show a very interesting pattern that proved to be highly significant for all the muscles measured.

Our data clearly indicate that women have a denser muscle cell structure (higher Z and R), and that women's muscle cells at rest operate with a higher metabolic activity (Ri), compared to that of men. This is supported by a lower Mc, indicating more membrane activity and possibly also a thinner membrane (Dodde et al. 2012; Tan et al. 2012), as supported by the observed higher center frequency (fc), suggesting a higher tension in women′s muscles at rest compared with those of men. This study has also revealed that the extracellular resistance (Re) was higher for women than for men. This would suggest that women′s muscles are not only denser, but there is less fluid around the cellular matrix, compared to men's muscles. This would cause a slower removal of waste products (i.e. lactate) and accordingly a slower transport of nutrients (i.e. glucose) from the blood to the active muscle cells. The lower PA, which is a combination of the Xc parameter divided by the R parameter, follows the trend observed for these two parameters, such that the PA is lower for women than for men. Women′s muscles being at rest more energetically active (higher Ri) fits well with the observations of a higher R and Re, and a lower Mc. How then are these findings integrated with what is currently understood about muscle and gender differences?

Looking at anatomical differences between men and women, Edama and colleagues (2017) showed gender‐based differences in the attachment and position of the muscle and fascia on the tibia. This most likely explains some of the reason for the higher incidence of medial tibial stress syndrome in women athletes compared with men. Another study (Otsuka et al. 2018) showed gender‐specific differences in the morphological as well as the mechanical properties of the fascia lata. These authors noted at the medial site, that the distribution of the fiber direction and the elastic properties of the fascia lata were gender‐specific, with women showing more elastic fascia (Otsuka et al. 2018). Combining these findings with our mfBIA results, one can extrapolate that women have stiffer muscle cells, but softer fascia, compared to men.

In the present study it was consistently shown that the fc was higher for women than for men for every muscle measured. It has previously been shown that treatment inducing muscle relaxation causes a decrease in the fc parameter (Elbrønd et al. 2015). Likewise, any change in cell size will have an effect on the membrane capacitance (Dodde et al. 2012).

Further support to the found gender difference can be found in a human cadaver study looking at the proportion of the longitudinal directed fibers in fascia lata showed that they were higher in men than in women (Otsuka et al. 2018). Moreover, the stiffness and the Young′s modulus of the fascia lata of females was higher in the transverse direction but lower in the longitudinal direction than that of males (Otsuka et al. 2018). Conversely, the medial site showed a higher longitudinal stiffness (N/mm) in females *cf* males. Although this study was carried out on formalin‐fixed cadavers, these differences must still hold. Together with the mfBIA results, this observation lends weight to the finding that women have a higher resting tension in their muscles in order to compensate for a more elastic fascia *cf* men.

### Importance of the present mfBIA data

The lack of age differences in mfBIA parameters seen in this study indicates that in a generally healthy population, the muscle cellular health is good despite a decrease in muscle strength with age (Danneskiold‐Samsoe et al. 2009). The gender difference found in this study clearly has implications for the frequency and intensity of the training performed by female as opposed to male athletes, which should be taken into consideration when designing training programs. Likewise, in all work situations (e.g nursing and care workers, heavy manual workers, computer users) the gender difference must be considered in terms of the long‐term loading on the musculoskeletal system. It must also be taken into account when designing rehabilitation programs and programs aimed at maintaining strength and balance in elderly subjects.

## Conclusion and perspectives

In healthy subjects, up to the age of 70 years, it is shown that the quality of skeletal muscles does not change despite some decrease in muscle strength. However, a clear gender difference in muscle tension and metabolic properties have now been revealed. This finding has implications for not only how to remain healthy and functional at work and with increasing age, but also in competitive sports and when designing programs for physical rehabilitation.

## Conflict of Interest

The authors know of no conflicts of interest in connection with this study.
